# Identification of novel small molecule inhibitors of centrosome clustering in cancer cells

**DOI:** 10.18632/oncotarget.1198

**Published:** 2013-09-25

**Authors:** Eiko Kawamura, Andrew B. Fielding, Nagarajan Kannan, Aruna Balgi, Connie J. Eaves, Michel Roberge, Shoukat Dedhar

**Affiliations:** ^1^ Department of Integrative Oncology, British Columbia Cancer Agency, Vancouver, BC, Canada; ^2^ current address: Department of Cellular and Molecular Physiology, Institute of Translational Medicine, University of Liverpool, Liverpool, UK; ^3^ Terry Fox Laboratory, BC Cancer Agency, Vancouver, BC, Canada; ^4^ Department of Biochemistry and Molecular Biology, University of British Columbia, Vancouver, BC, Canada; ^5^ Department of Medical Genetics, University of British Columbia, Vancouver, BC, Canada

**Keywords:** Centrosome Clustering, multipolar mitosis, small molecule, cancer specific, spindle

## Abstract

Most normal cells have two centrosomes that form bipolar spindles during mitosis, while cancer cells often contain more than two, or “supernumerary” centrosomes. Such cancer cells achieve bipolar division by clustering their centrosomes into two functional poles, and inhibiting this process then leads to cancer-specific cell death. A major problem with clinically used anti-mitotic drugs, such as paclitaxel, is their toxicity in normal cells. To discover new compounds with greater specificity for cancer cells, we established a high-content screen for agents that block centrosome clustering in BT-549 cells, a breast cancer cell line that harbors supernumerary centrosomes. Using this screen, we identified 14 compounds that inhibit centrosome clustering and induce mitotic arrest. Some of these compounds were structurally similar, suggesting a common structural motif important for preventing centrosome clustering. We next compared the effects of these compounds on the growth of several breast and other cancer cell lines, an immortalized normal human mammary epithelial cell line, and progenitor-enriched primary normal human mammary epithelial cells. From these comparisons, we found some compounds that kill breast cancer cells, but not their normal epithelial counterparts, suggesting their potential for targeted therapy. One of these compounds, N2-(3-pyridylmethyl)-5-nitro-2-furamide (Centrosome Clustering Chemical Inhibitor-01, CCCI-01), that showed the greatest differential response in this screen was confirmed to have selective effects on cancer as compared to normal breast progenitors using more precise apoptosis induction and clonogenic growth endpoints. The concentration of CCCI-01 that killed cancer cells in the clonogenic assay spared normal human bone marrow hematopoietic progenitors in the colony-forming cell assay, indicating a potential therapeutic window for CCCI-01, whose selectivity might be further improved by optimizing the compound. Immunofluorescence analysis showed that treatment with CCCI-01 lead to multipolar spindles in BT-549, while maintaining bipolar spindles in the normal primary human mammary epithelial cells. Since centrosome clustering is a complex process involving multiple pathways, the 14 compounds identified in this study provide a potentially novel means to developing non-cross-resistant anti-cancer drugs that block centrosome clustering.

## INTRODUCTION

Bipolar spindle formation is crucial for correct segregation of duplicated chromosomes into two daughter cells during cell division. Centrosomes are microtubule organizing centers and serve as poles of spindles during mitosis. In most animal cells, there are only two centrosomes that form two poles of a bipolar spindle during mitosis. In contrast, many cancer types are known to contain more than two or “supernumerary” centrosomes [1, 2]. Supernumerary centrosomes can result in extra spindle poles, which in turn could lead to multipolar cell division with significant chromosome mis-segregation or cell cycle arrest, followed by cell death [3, 4]. To avoid detrimental multipolar divisions, cancer cells use mechanisms to assemble centrosomes into two functional poles so that bipolar spindles can be formed [5]. This process is called centrosome clustering. Genome-wide RNAi screens carried out in *Drosophila* S2 cells and a human oral cancer cell line revealed a large number of pathways and genes involved in centrosome clustering [6, 7]. Various molecular regulators for clustering dependent adaptation process have been identified and include motor proteins, centrosomal proteins, kinetochore proteins, spindle assembly checkpoint proteins, sister chromatid cohesion proteins, chromosomal passenger complex members, microtubule associated proteins and components of the actin cytoskeleton [5-8].

While microtubule-targeting anti-mitotic drugs are important components of many cancer chemotherapy regimens, these drugs also hinder mitosis and alter microtubule dynamics in normal cells leading to adverse side effects such as myelosuppression, neurotoxicity, gastrointestinal symptoms and alopecia [9]. Since supernumerary centrosomes are common in cancer cells but not in healthy cells, targeting centrosome clustering has been suggested as a strategy to obtain greater cancer-specificity [10, 11] and recent studies have shown that blocking centrosome clustering can be effective in killing cancer cells, while sparing normal cells *in vitro* [6, 8, 12, 13] and *in vivo* [13]. An anti-fungal agent, Griseofulvin, which binds to tubulins [14-16] and shows anti-tumor activity [17], was identified in a fungal extract library screen for molecules that inhibit centrosome clustering [12]. We have previously shown that QLT-0267, which is an inhibitor of the focal adhesion and centrosomal protein, integrin-linked kinase (ILK) [18, 19], is another compound that can inhibit centrosome coalescence [8]. The discovery of structurally different molecular regulators of this process suggests possible additional opportunities to identify cancer cell-specific druggable targets with reduced undesirable side effects.

In this study, we carried out a high-content screen of a chemical library composed of pure drug-like compounds to discover novel small molecules that inhibit centrosome clustering in cancer cells. Through our screen, we identified 14 new active compounds, which were further examined for their cytotoxicity in cancer and normal cells. N2-(3-pyridylmethyl)-5-nitro-2-furamide, which we have named Centrosome Clustering Chemical Inhibitor-01 (CCCI-01), showed the most promising differential effects between cancer and normal cells. CCCI-01 treatment resulted in multipolar spindles in nearly 90% of BT-549 cells, while freshly isolated normal primary human mammary epithelial cells (HMEC) maintained bipolar spindles. These findings demonstrate the utility of this approach to the development of a new type of cancer-specific therapeutics and for advancing our knowledge of the biological functions of genes required for mitosis.

## RESULTS

### High-content screen to identify small molecules that inhibit centrosome clustering in cancer cells with supernumerary centrosomes

We developed a cell-based high-throughput screen to discover small molecules that can block centrosome clustering using the human BT-549 breast cancer cell line as the testing platform. BT-549 cells were chosen because they contain supernumerary centrosomes that cluster into two poles to form bipolar spindles when they divide [6, 8]. A chemical collection consisting of > 5,000 small molecules with drug-like structures was screened. Cells were incubated in 96-well plates overnight, exposed to each test compound at a final concentration of approximately 17 μM for five to seven hours, and then fixed with paraformaldehyde. Cells were then labeled with TG-3, a monoclonal antibody that recognizes phosphorylated form of nucleolin that peaks during mitosis and therefore is a marker for mitosis [20, 21], anti-pericentrin to visualize the centrosomes and Hoechst 33342 to stain the DNA. Images were automatically acquired from three channels (to detect Hoechst, TG-3 and pericentrin) per field and 15 fields per well of each 96-well plate using a Cellomics Array Scan VTI microscope.

**Figure 1:**
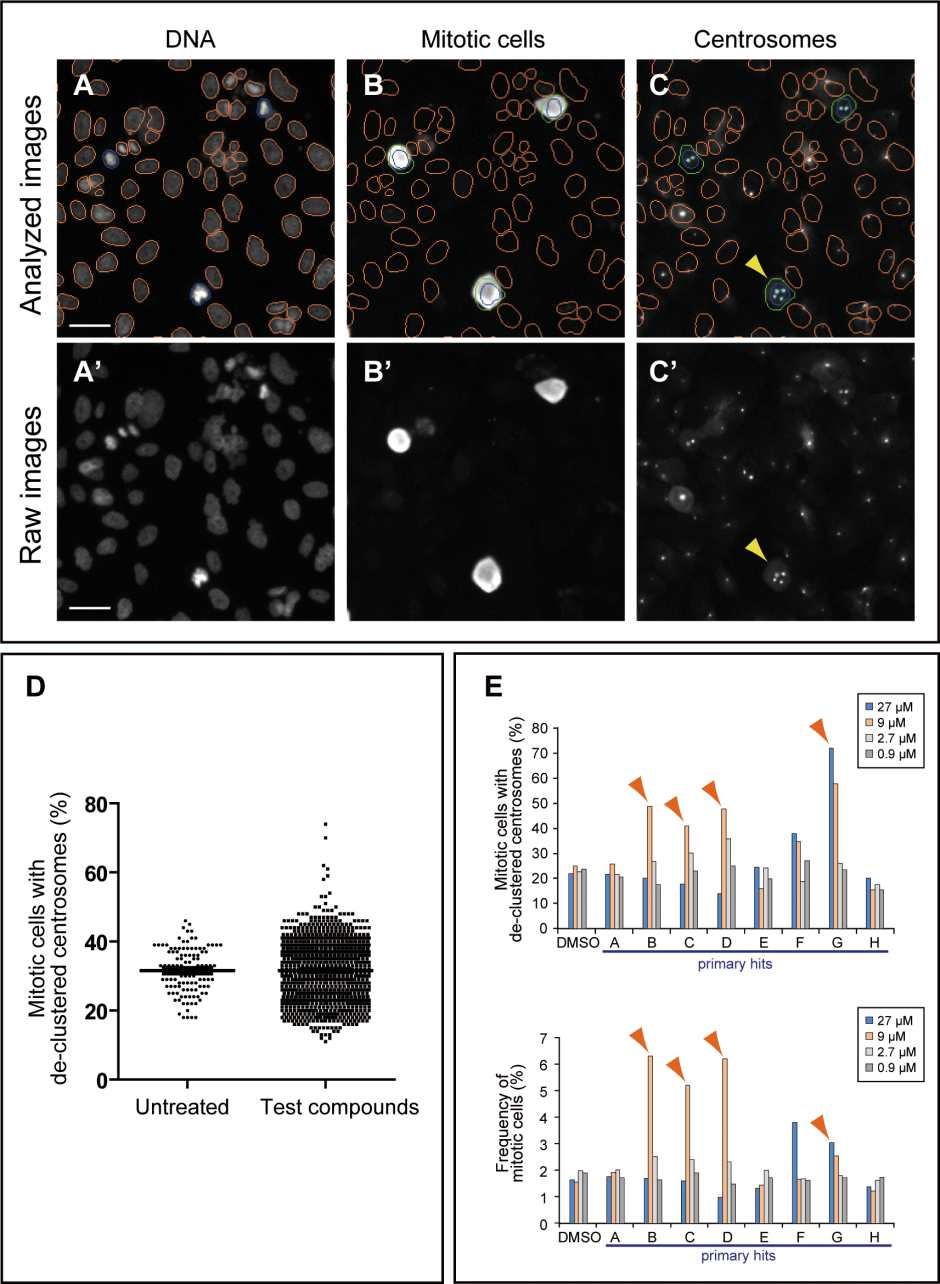
High-content screening strategy for the identification of candidate compounds that inhibit centrosome clustering (A-C) Analysis of BT-549 cells for de-clustered centrosomes using a Cellomics Array Scan VTI imaging platform with Thermo Scientific Compartmental Analysis algorithm. (A and B) Identification of nuclei/cells and mitotic cells. Cells were labeled with Hoechst 33342 (A) and TG-3, a marker of mitotic cells (B). The nuclei of TG-3-positive (mitotic) cells are outlined in blue. TG-3-negative (non-mitotic) cells are outlined in orange. A border was introduced by expanding 5 pixels outward from the nuclear boundary of a TG-3 positive cell to define a region of interest (ROI, green circles) (B). (C) Identification of centrosomes in mitotic cells. Centrosomes were labeled with anti-pericentrin (light blue dots) and the number of pericentrin foci within the ROIs was enumerated by the software. Cells with de-clustered centrosomes were defined as those mitotic cells with greater than two pericentrin foci. Bar = 50 μm. (A'-C') Corresponding raw images before compartmental analysis. Bar = 50 μm. (D) Representative results of a primary screen evaluating 1,200 compounds. The distribution of mitotic cells with de-clustered centrosomes for the test group is similar to that for the untreated group except that it displays additional high scores. Those compounds that increased the score by at least 2.5x the standard deviation were considered “hits” and were subjected to a secondary screen. (E) Representative results of a secondary screen. Four different concentrations (27, 9, 2.7 and 0.9 μM) were tested for each primary hit. The frequency of cells with de-clustered centrosomes and mitotic indices are shown for primary hits, A-H, and negative DMSO control. Arrowheads indicate hits in the secondary screen. Compound F was a false positive due to fluorescent debris present in the well.

For automated data analysis, the Thermo Scientific Compartmental Analysis algorithm was employed (Methods) (Figure 1A-C). The total number of cells was obtained by enumerating nuclei in the Hoechst channel (orange or blue nucleus outline; Figure 1A and A'). The total number of mitotic cells was obtained by counting TG-3 positive cells (green circles, Figure 1B) in the TG-3 channel. The mitotic index (percentage of cells in mitosis) was calculated based on the total number of cells and TG-3 positive mitotic cells. Mitotic cells with more than two centrosome foci detected in the pericentrin channel were considered to have de-clustered centrosomes (arrowheads, Figure 1C and C'). The percentage of mitotic cells with de-clustered centrosomes was calculated by dividing the number of mitotic cells with de-clustered centrosomes by the total number of mitotic cells. Eight wells of each 96-well plate received no chemicals and were treated as negative controls. The distribution for the frequency of mitotic cells with de-clustered centrosomes in a test group was similar to that for negative control, except for some wells showing higher scores (representative results of a primary screen; Figure 1D). Test compounds scoring higher than the plate average + 2.5 × the standard deviation were considered as hits. When a score was near this cut-off value, the mitotic index was taken into consideration, since centrosome de-clustering often induces mitotic arrest [6-8, 12]. Out of 5,440 compounds tested in primary screens, we identified 88 (1.6%) primary hits (Table 1). To confirm their activity, positive compounds were tested in a dilution series (0.9, 2.7, 9.0 and 27 μM) in duplicates in the same assay. A result of such a secondary screen is shown in Figure 1E as an example. In this case, compounds B, C, D and G were considered as active compounds, since they increased the frequency of mitotic cells with de-clustered centrosomes. These four compounds also induced mitotic arrest (Figure 1E). In addition to the automated analysis, all images from hits were visually examined to ensure that staining or imaging artifacts were excluded. For instance, compound F in Figure 1E indicated higher de-clustering and mitotic index, but these were due to fluorescent debris present in the well. Out of 88 primary hits examined, 18 compounds were confirmed as active in secondary screens. We further examined these small molecules (in total of 17 compounds as one compound was no longer available) in detail under standard culture conditions.

**Table 1:** Summary of the high-content screen

	number of compounds
total number of compounds tested in primary screens	5440
hits from primary screens	88 (1.6%)
hits after secondary screens	18 (0.3%)
secondary hits tested in standard culture condition	17
confirmed hits after testing in standard culture condition	14

### Immunofluorescence microscopy analysis of active compounds in standard culture conditions

**Figure 2:**
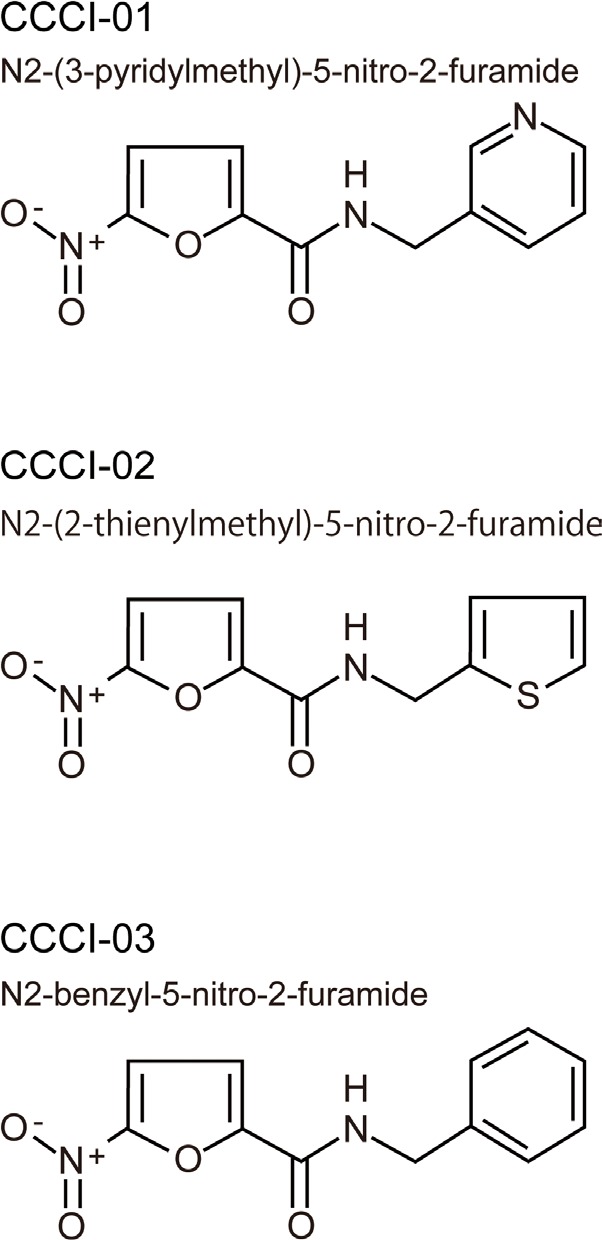
Chemical structures of three similar hits Three active compounds that share structural similarities

We compared structures of active compounds and identified three compounds (named CCCI-01, 02 and 03, Figure 2) that shared structural similarity. Another eight related small molecules (Supplemental Figure 1) did not prevent centrosome clustering in the primary screen. The 5-nitro-2-furamide moiety, circled in blue (Supplemental Figure 1), is also present in some inactive compounds, indicating that this group alone is not sufficient to prevent centrosome clustering. The three active compounds have a 5- or 6-membered ring separated from the 5-nitro-2-furamide by an extra carbon while inactive compounds did not have this extra carbon, indicating that the spacing between the two ring systems is important for activity. These inactive compounds will be useful for future detailed structure-activity relationship analyses.

**Figure 3:**
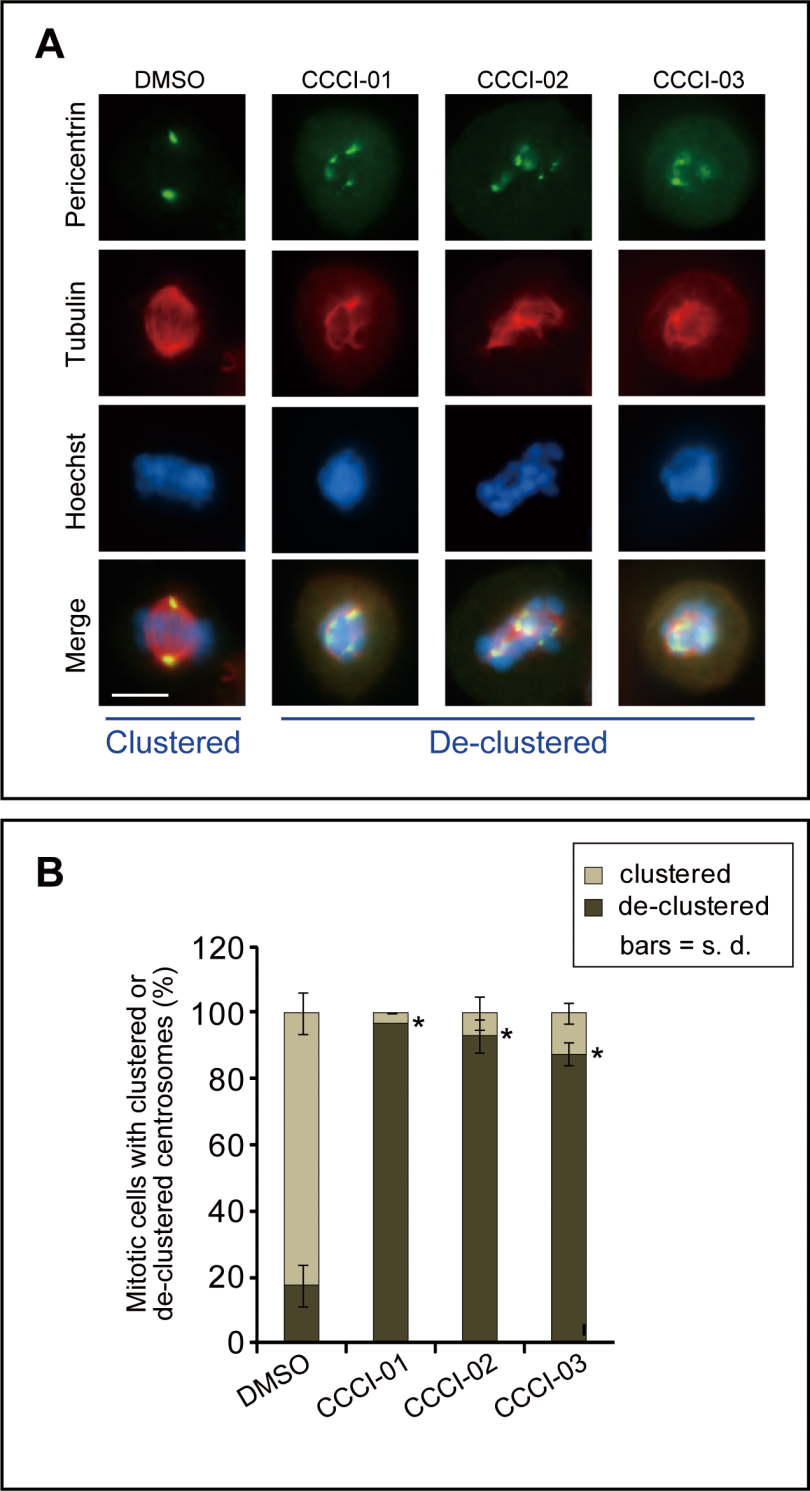
Effects of identified small molecules on spindle organization *in vitro* (A) Representative immunofluorescence images of BT-549 cells showing the centrosome de-clustering properties of the positive compounds identified in the high-content screen. Cells were treated with the various compounds for 5 hours at a concentration of 10 μM, and then immunolabelled for pericentrin (green) and tubulin (red). Nuclei were labeled with Hoechst 33342 (blue). Bar = 10 μm. (B) Quantification of the frequency of mitotic cells with de-clustered centrosomes after treatment as described in (A). Centrosome de-clustering was analyzed using pericentrin labeling, and tubulin labeling was also used to ensure multipolar spindle formation. At least 25 mitotic cells/treatment/experiment were measured for each compound tested. Average of at least two independent experiments is shown. *P < 0.03, student's t-test.

All active compounds were examined for spindle polarity, centrosome clustering and chromosome alignment in BT-549 cells grown in a standard 6-well format. Cells were treated with 10-30 μM of compounds for five hours and then subjected to immunofluorescence microscopy for tubulin and pericentrin. DMSO-treated cells served as negative controls and generally formed morphologically normal bipolar spindles with well defined metaphase plates (Figure 3A). Treatment with 10 μM of CCCI-01, 02 and 03, shown as examples, increased the incidence of de-clustered centrosomes with multipolar spindles and highly disorganized chromosome arrangements (Figure 3A). Quantitative analysis of centrosome de-clustering was carried out according to Fielding *et al*. [8]. The results showed that the three similar compounds (Figure 2) increased the frequency of mitotic cells with de-clustered centrosomes from 15% to about 90% (*P* < 0.03, Figure 3B). In the primary and secondary screens, all three compounds, CCCI-01, 02 and 03, arrested cells in mitosis (Figure 1E, compounds B, C and D, respectively; data not shown for the primary screen).

Out of 17 active compounds examined, 14 compounds were confirmed to prevent centrosome clustering and induce multipolar spindles and disorganized chromosome alignment (data not shown). Interestingly, the compounds that did not increase mitotic indices turned out to be false positives, confirming previous reports that interference with centrosome clustering often results in prolonged mitosis [6-8].

### Effects of active compounds on viability in cancer and normal cells

**Figure 4:**
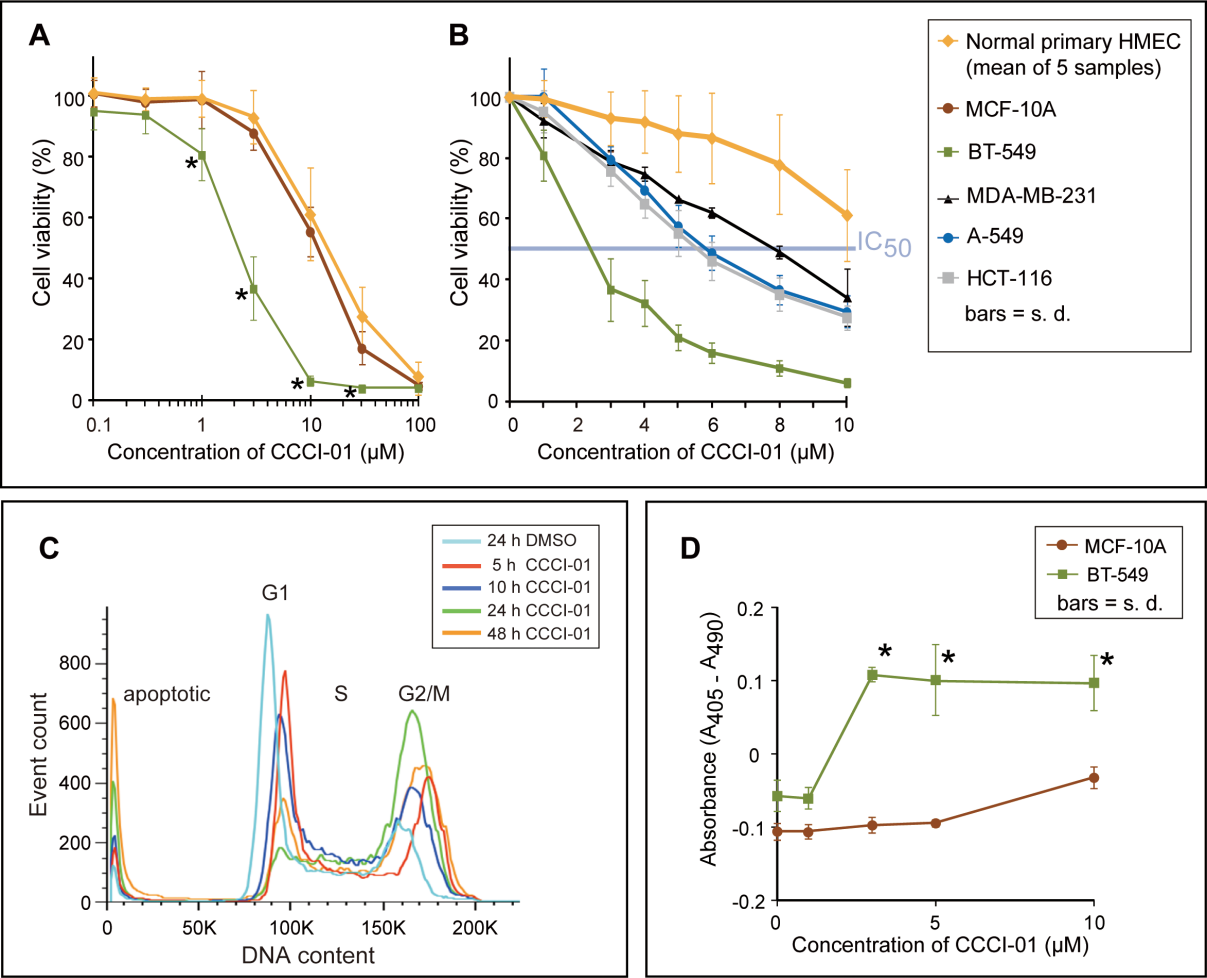
Comparison of cytotoxicity of CCCI-01 in cancer cells and normal cells (A) Cell viability in cancer and normal cells was examined after 2 days of incubation with CCCI-01 by MTT assay. BT-549 was more sensitive to CCCI-01 compared to five primary normal human mammary epithelial cells (HMECs, passage 0) from five different reduction mammoplasty samples and MCF-10A, an immortalized normal mammary epithelial cell line. *P < 0.005 (compared to normal cells), student's t-test. (B) MTT assay was carried out with finer titration steps between 1 and 10 μM. In addition to BT-549, MDA-MB-231, A549 and HCT-116 were also examined to compare cytotoxicity in different types of cancer. IC_50_ values for cancer cells were between 2 and 8 μM, while that for primary HMEC was above 10 μM. *P* < 0.001 for BT-549 at 1-10 μM; *P* = 0.045 and *P* < 0.005 for MDA-MB-231 at 1 μM and 3-10 μM, respectively; *P* < 0.02 for A-549 and HCT-116 at 3-10 μM, student's t-test, compared to primary HMEC. (C) Time course analysis of cell cycle and cell death by flow cytometry was carried out in BT-549 treated with 5 μM CCCI-01. Mitotic arrest represented by the increased G2/M population was observed with CCCI-01 treatment, while the G1 population decreased. Apoptosis, the sub-G1 population, was elevated in the presence of CCCI-01 with prolonged treatment. The distribution of S phase population remained similar, indicating there were no significant effects on DNA synthesis by CCCI-01. DMSO had essentially no effects on these peaks (Supplemental Figure 3). A representative result from three independent experiments is shown. (D) Apoptosis was examined by Cell Death Detection ELISA (Roche). BT-549 and MCF-10A were treated with CCCI-01 or DMSO for 19 hours. Values were normalized (= 0) to the highest DMSO concentration (0.1 %) applied for CCCI-01 treatment. The level of apoptosis was increased in BT-549 as low as at 3 μM, while apoptosis was not induced in MCF-10A even at 10 μM. This assay was carried out three times, and produced consistent results. A representative result is presented. *P < 0.01, student's t-test.

In order to compare the effects of the inhibitors of centrosome clustering on cancer versus normal breast cells, we carried out the MTT assay to measure cell yields after two days of compound treatment. As representative of normal cells, five primary normal human mammary epithelial cells (HMEC; passage 0) isolated by fluorescent activated cell sorting (FACS) from five different reduction mammoplasty samples, and MCF-10A cells which are widely used as an immortalized “normal” human breast cell line were tested. A dilution series of CCCI-01 ranging from 0.1 to 100 μM was examined. As shown in Figure 4A, CCCI-01 inhibited the viability of BT549 breast cancer cells at much lower concentrations compared to normal epithelial cells. Accordingly, we examined its effects in greater detail. To assess its effects on other different tumor cell types, we extended our studies to MDA-MB-231 cells, another breast cancer cell line, A-549, a lung cancer cell line, and HCT-116, a colon cancer cell line. IC_50_ values for primary HMEC and normal MCF-10A were greater than 10 μM, while that for BT-549 was less than 3 μM, providing a greater than 3-fold therapeutic window in this *in vitro* assay (Figure 4A and B). In general, CCCI-01 more effectively inhibited the growth of various cancer cells compared to the normal cells. Interestingly, the degree of cytotoxicity varied among different cancer cell lines (IC_50_ for BT-549, 3 μM; MDA-MB-231, 8 μM; A-549 and HCT-116, 6 μM), indicating involvement of different mechanisms for the survival of different cancer cells.

Each active compound validated from our screen was tested on primary normal HMEC (passage 0) from at least two different reduction mammoplasty samples and two breast cancer cell lines, BT-549 and MDA-MB-231. All of the 14 active compounds reduced cell viability in a concentration-dependent manner (Supplemental Figure 2 for CCCI-01, 02 and 03; unpublished data for other compounds) while DMSO treatment had little or no effect on the yield of any of the cells tested in this assay (Supplemental Figure 2). However, of these 14 compounds, CCCI-01, 02 and 03 showed the most differential effects on the viability of various cancer cell lines and the normal cells (Supplemental Figure 2). Of these three nitrofuramide compounds, CCCI-01 showed the greatest differential response.

### Effects of CCCI-01 on cell cycle and viability of BT-549 cancer cells

To examine further properties of CCCI-01 on cell viability, we analyzed cell cycle and cell death in BT-549 by flow cytometry. Cells were treated with 5 μM of CCCI-01 and were examined for their DNA content over a period of time (Figure 4C). CCCI-01 treatment increased the number of cells in G2/M phase compared to the DMSO control. This is consistent with our screening data that showed mitotic arrest by CCCI-01 treatment with increased TG-3 labeling, a marker for mitotic cells (Compound B in Figure 1E is CCCI-01). The S phase population seemed unaffected, indicating that CCCI-01 does not block DNA synthesis. The sub-G1 population, which represents apoptotic cells, increased with prolonged CCCI-01 treatment, while DMSO had no effects on this peak (Supplemental Figure 3). These results demonstrate that CCCI-01 reduced viability of BT-549 by combination of mitotic arrest and cell death.

### Induction of apoptosis by CCCI-01 in BT-549 cells but not in normal MCF-10A cells

To analyze selective cytotoxicity of CCCI-01 against cancer, we next compared induction of apoptosis in cancerous BT-549 and normal MCF-10A using Cell Death Detection ELISA (see Methods) which measures fragmented nucleosomes in the cytoplasm associated with apoptosis. After 19 hours of incubation with 3 μM of CCCI-01, the level of apoptosis was significantly elevated in BT-549 cells (Figure 4D), without further increases with higher concentrations. In contrast, in MCF-10A, apoptosis was not increased even at 10 μM. These data demonstrate selective induction of apoptosis in breast cancer cells relative to normal mammary epithelial cells, likely due to failure of bipolar mitosis resulting from centrosome de-clustering.

### Selective inhibition of colony formation by CCCI-01

**Figure 5:**
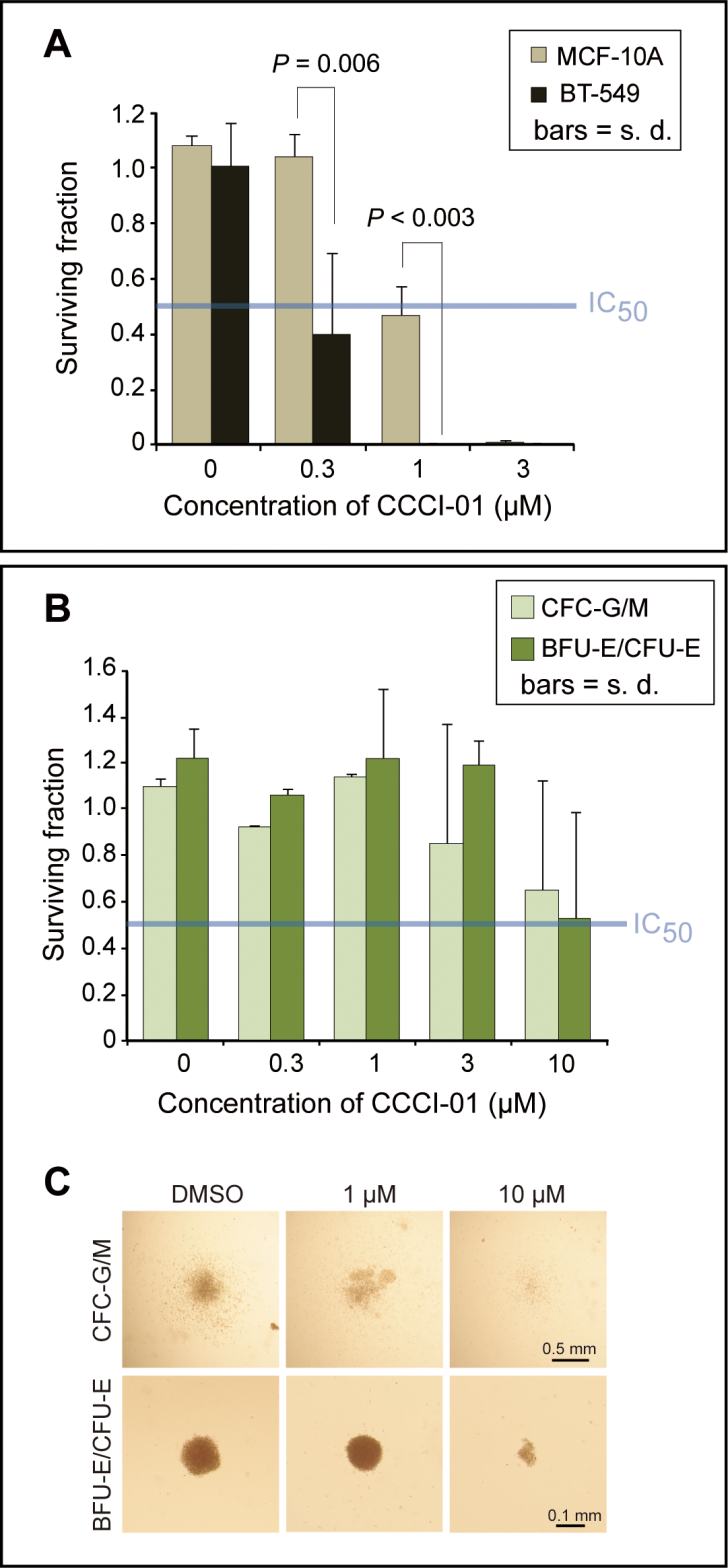
Effects of CCCI-01 in colony formation of cancer and normal cells (A) Clonogenic assay was carried out in BT-549 and MCF-10A treated with CCCI-01. Colonies consisting of more than 50 cells were counted after 9 days of incubation with CCCI-01 or DMSO. All treatments received 0.06 % DMSO. Surviving fraction was obtained by normalizing to DMSO control (= 1). Values are average from at least two independent experiments. *P*, student's t-test. The blue line in the graph indicates IC_50_. (B and C) The CFC assay was carried out in primary normal bone marrow cells. Cells were cultured in the presence of varying concentrations of CCCI-01. (B) Surviving fractions of CFC-G/M and BFU-E/CFU-E colonies were obtained by normalizing to the DMSO control (= 1). Values are average of two independent experiments with two normal bone marrow samples: one is nomonuclear cells and the other is selected for CD34+. Separate data are available in Supplemental Table 1. For CFC-G/M, no inhibitory effects were detected up to 1 μM, and colony size and number were reduced in both samples at 10 μM. BFU-E/CFU-E colonies did not seem to be affected up to 3 μM, and showed inhibition in colony formation at 10 μM in both samples. The blue line in the graph indicates IC_50_. (C) Representative images of each type of colonies treated with different concentrations of CCCI-01.

Next, we compared the effects of CCCI-01 on BT-549 cancer cells and MCF-10A normal cells in clonogenic assays [22]. Single cells were plated and cultured for 9 days with varying concentrations of CCCI-01 (Figure 5A). At 0.3 μM, BT-549 colony formation was reduced by 60 % (*P* = 0.03), while MCF-10A colony yields were unaffected (*P* = 0.48, Figure 5A). When the concentration was increased to 1 μM, colony formation by BT-549 cells was completely inhibited. In contrast, colony formation by MCF-10A cells was reduced by only 60 % (*P* < 0.003, BT-549 vs. MCF-10A). This shows that CCCI-01 selectively inhibits the ability of BT-549 clonogenic cells to undergo more than five divisions as compared to normal MCF-10A cells.

### Poor cytotoxicity of CCCI-01 in normal human hematopoietic progenitor cells from bone marrow isolates

Immunosuppression is a common dose-limiting side effect in chemotherapy. The colony-forming cell (CFC) assay of normal bone marrow cells is often used to predict the severity of myelosuppression caused by inhibition of hematopoietic progenitor activity [23]. Therefore, we examined cytotoxic effects of CCCI-01 in the CFC assay using primary normal bone marrow cells. Due to limited availability of normal bone marrows, CCCI-01 was tested on samples from two healthy donors: one was selected for hematopoietic progenitors (CD34 positive cells) and the other was unselected (Figure 5B and C, separate data available in Supplemental Table 1). Cells were plated in methylcellulose-based media (STEMCELL Technologies, Vancouver, Canada) with varying concentrations of CCCI-01 and colonies were evaluated after culturing for 15 or 16 days. Up to 1 μM of CCCI-01, no changes in the number and size of CFU-G/M colonies were detected in both samples. At 3 μM, reduction in the CFU-G/M colony number was observed in one sample but not in the other. At 10 μM, proliferation of CFU-G/M was inhibited in both samples. The number of BFU-E/CFU-E colonies was not affected up to 3 μM, and reduction was observed at 10 μM. IC_50_ values for CFU-G/M and BFU-E/CFU-E were approximately 10 μM when averaged. Considering IC_50_ of CCCI-01 for BT-549 in clonogenic assay was below 0.3 μM, CCCI-01 may exert minimal toxic effects to normal myeloproliferative cells in the concentration ranges that are sufficient to inhibit the growth of cancer cells.

### No induction of multipolar spindle in primary human mammary epithelial cells purified from reduction mammoplasties

**Figure 6:**
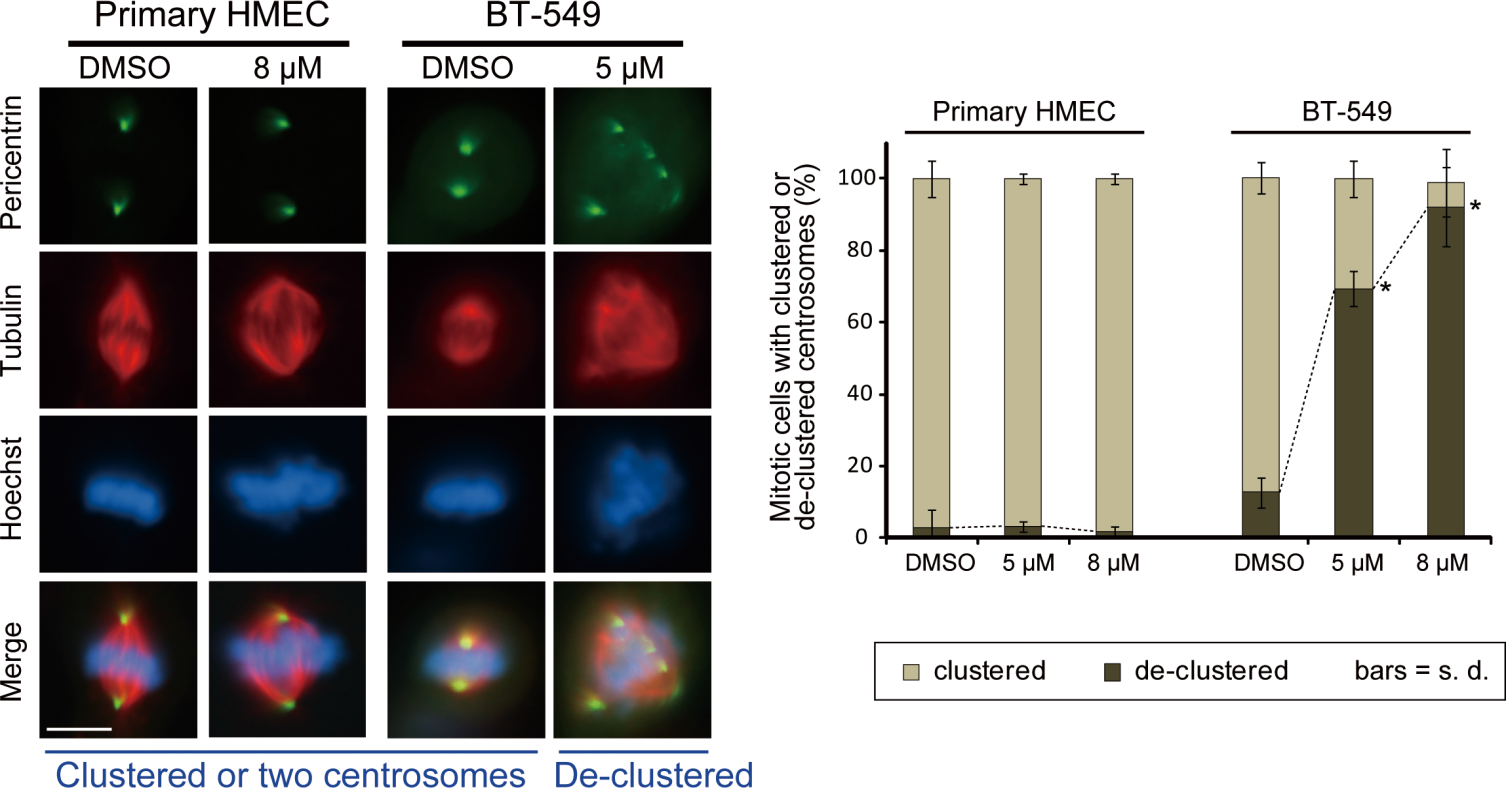
Effects of CCCI-01 on spindle polarity in BT-549 cancer cells and normal primary HMECs Centrosome arrangement and spindle multipolarity were examined by immunofluorescence after treating cells with CCCI-01 for 5 hours. As little as 5 μM increased centrosome de-clustering to 70 % in BT-549 breast cancer cells (average of two independent experiments, at least 30 mitotic cells/treatment/experiment). In contrast, even at 8 μM, de-clustering was not induced (*P* > 0.7, student's t-test) in normal primary HMEC from three different reduction mammoplasty samples (average of two independent experiments with three HMEC samples in total, at least 30 mitotic cells/treatment/sample). Scale bar = 10 μm. *P < 0.02, student's t-test.

Although normal cells contain only two centrosomes, it is possible that CCCI-01 induces fragmentation of normal centrosomes resulting in multipolar spindle formation. Therefore immunolabeling was performed to evaluate the spindle and centrosome arrangement in normal primary HMECs treated with 5 or 8 μM of CCCI-01. BT-549 cells were also tested to see if these lower concentrations can interfere with centrosome organization during mitosis. Even at 8 μM, the higher concentration tested, most spindles were bipolar with two poles labeled by anti-pericentrin, and chromosomes were aligned at the metaphase plate in primary HMECs (passage 0) isolated from three different reduction mammoplasty samples (Figure 6). As expected, for the rare mitotic primary normal cells that did form multipolar spindles, this was not specific to either DMSO or CCCI-01 treatment (*P* > 0.7). In contrast, 5 μM CCCI-01 was sufficient to induce multipolar spindles in BT-549 cells (Figure 6). The frequency of BT-549 cells with de-clustered centrosomes was increased from a base value of 17 %, observed in the negative control, to 68 % and 86 % in the presence of 5 μM and 8 μM CCCI-01, respectively (*P* < 0.02). This shows that treatment with CCCI-01 leads to multipolar spindles in BT-549 cells, but not in primary normal cells which are unlikely to rely on centrosome clustering for bipolar spindle formation.

### Analyses of inactive versus active compounds

**Figure 7:**
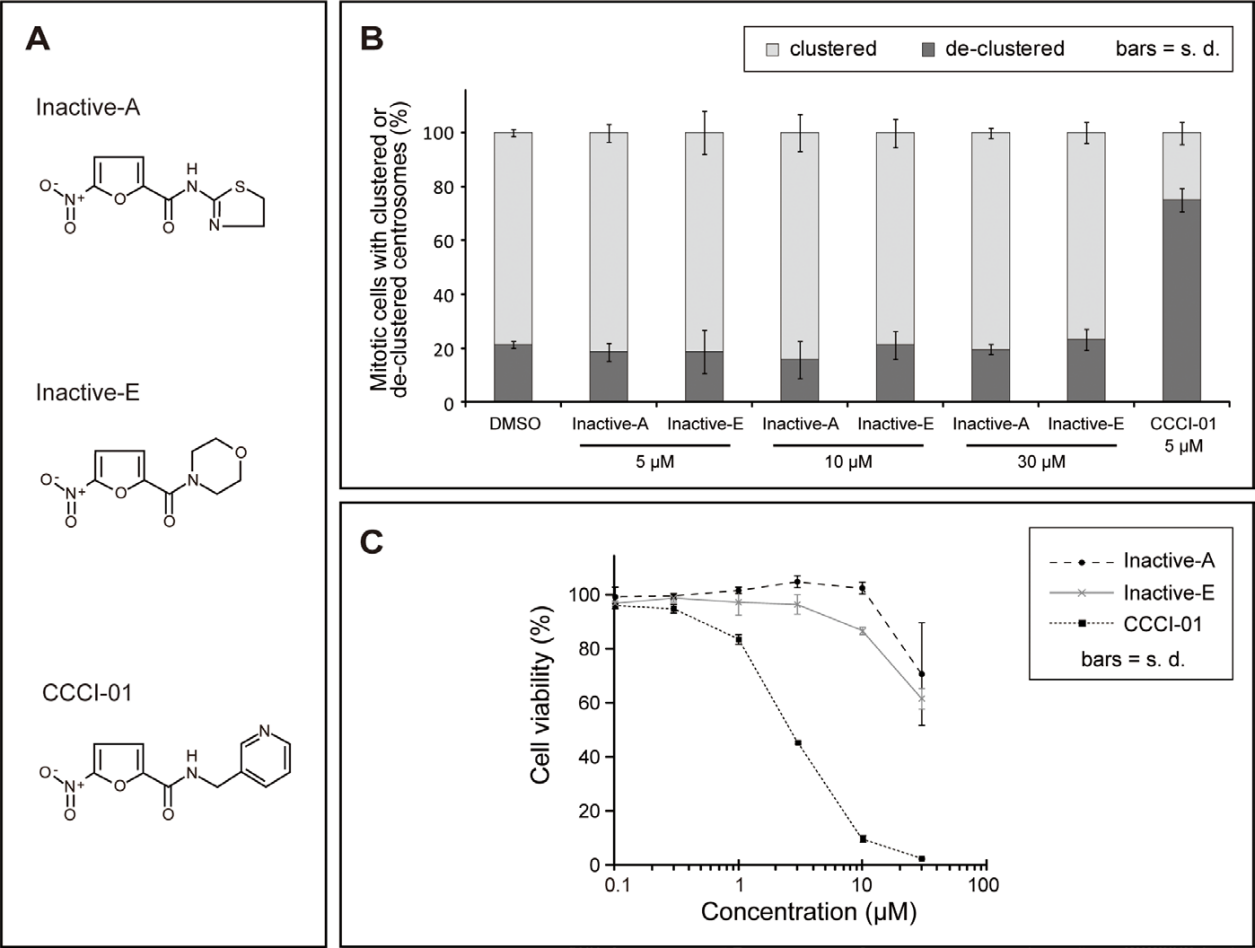
Analysis of inactive compounds structurally similar to CCCI-01, 02 and 03 (A) Chemical structures of two inactive compounds, Inactive-A and E, and active CCCI-01. (B) Centrosome arrangement and spindle multipolarity were examined by immunofluorescence after treating cells with Inactive-A or E or CCCI-01. Inactive-A and E did not inhibit centrosome clustering even at 30 μM, while as low as 5 μM of CCCI-01 resulted in 70% of mitotic cells with de-clustered centrosomes. Only the highest concentration of DMSO (0.6 %) applied is presented as a negative control here. All corresponding DMSO showed no detectable effects on the spindle organization. Average from three independent experiments (at least 42 mitotic cells/treatment/experiment). (C) Cell viability in BT-549 was examined after 2 days of incubation with Inactive-A or E or CCCI-01 by the MTT assay. The cell viability in BT-549 was not affected by Inactive-A and E up to 10 μM, while at this concentration, CCCI-01 reduced the cell viability to less than 10 %. At 30 μM, Inactive-A and E showed some cytotoxicity, but this is not caused by centrosome de-clustering as centrosomes did not display scattered configuration at this concentration (see Figure 6B). Average of three independent experiments.

To narrow down active groups in CCCI-01, 02 and 03, we examined two analogues, Inactive-A and E (Figure 7A), that appeared inactive from the primary screen (Supplemental Figure 1). First, their effects on spindle formation were assessed by immunefluorescence microscopy in BT-549 cells treated with compounds for 5 hours. While 5 μM of CCCI-01 was sufficient to increase the frequency of mitotic cells with de-clustered centrosomes to over 70 %, Inactive-A and E did not lead to fragmentation or de-clustering of centrosomes even at 30 μM (Figure 7B), confirming the result of the primary screen. If these inactive compounds exhibit minimal toxicity to BT-549, it implies that the cytotoxicity caused by CCCI-01, 02 and 03 was due to multipolar spindle formation with de-clustered centrosomes. Therefore we analyzed the cell viability in BT-549 cells by MTT assays. After two days of compound treatment, the inactive compounds did not show cytotoxicity even at 10 μM, however CCCI-01reduced the cell viability to 10 % at 10 μM. When the concentration was increased to 30 μM, some cytotoxicity was detected in Inactive-A or E treated cells, but this was not caused by centrosome de-clustering as centrosomes and spindles appeared unaffected at this concentration (Figure 7B). These results suggest that CCCI-01, 02 and 03 reduce viability of BT-549 cells through inhibition of centrosome clustering. Our structure-activity relationship analysis suggests that the 5-nitro-2-furamide (circled in blue in Supplemental Figure 1), a common structure in these active and inactive compounds, alone is not sufficient for inhibiting centrosome clustering and that the spacing between the 5-nitro-2-furamide and a 5- or 6-membered ring may be important for their activity.

## DISCUSSION

Targeting molecular processes that are selectively required by cancer cells to maintain their survival but not by normal cells, offers an ideal strategy to retain a therapeutic effect while reducing or eliminating side effects. There are only two centrosomes in normal cells during mitosis, whereas supernumerary centrosomes are very common in solid and hematological tumors [1]. Targeting the ability of supernumerary centrosomes to coalesce selectively inhibits cancer cell proliferation *in vitro* [6, 8, 12], suggesting centrosome clustering could be an attractive target for cancer-specific therapy. Here we carried out a high-content screen and identified 14 novel compounds that inhibited centrosome clustering. Some of these compounds were analyzed for their ability to act on centrosomes during mitosis and for their cytotoxicity in cancer and primary normal cells. These analyses lead to the identification of a class of nitrofuramide compounds represented by three distinct small molecules which show a differential killing between cancer and normal cells.

### Possible dependence of different cancer cells on different mechanisms to cluster centrosomes

Genome-wide RNAi screens in *Drosophila* and human cells revealed multiple pathways required for centrosome clustering [6, 7]. The MTT assay of active compounds revealed a variable degree of cytotoxicity among different cancer cell lines. For instance, BT-549 cells are more sensitive to CCCI-01 than MDA-MB-231 cells (Figure 4B), whereas BT-549 cells appear less sensitive to a structurally distinct active compound, as compared to MDA-MB-231 cells (unpublished data) in spite of the fact that approximately 45 % of cells in both lines contain extra centrosomes [6]. Thus the frequency of cells with extra centrosomes is not the sole determinant of the degree of cytotoxicity of these compounds in human cancer cells, suggesting different mechanisms are involved in clustering supernumerary centrosomes in different cancer cell types, even when they originate from the same tissue. Further characterization of the various classes of compounds identified here may help to elucidate the nature of these mechanisms and improve the development of this approach to selectively target cancer cells.

### Centrosome de-clustering and centrosome fragmentation

True centrosomes contain a pair of centrioles surrounded by pericentriolar material, PCM, while acentriolar centrosomes lack centrioles and can be formed by fragmentation of PCM [1, 24]. About half of BT-549 cells are known to contain extra true centrosomes [6]. Many of our active compounds showed higher than 50% of de-clustered mitotic cell population. This indicates that some multipolar spindle poles were anchored to acentriolar centrosomes. Such defects are known to be induced by knockdown of Aurora-A [25], an extensively studied oncogene involved in multiple steps of mitosis [26], and depletion of Cep57 [27], a centrosomal protein with microtubule bundling activity [28]. It has been suggested that acentrosomal and centrosomal poles need to be clustered to assemble bi-polar spindles in cancer cells with normal number of bona fide centrosomes [29]. This pole focusing mechanism is dependent on HSET, a kinesin involved in centrosome clustering in cancer cells but is dispensable in normal cells [6, 29]. It is also possible that these compounds cause abnormal centriole splitting leading to a centrosome with only one centriole instead of a pair of centrioles. Abnormal centriole disengagement accompanied by multipolar spindle formation has been reported for RNAi of Astrin [30] or Akt kinase–interacting protein 1 [31], which are both important for the maintenance of centrosome integrity. Another possibility is that cells with de-clustered centrosomes were effectively arrested in mitosis, resulting in a selective accumulation of such mitotic cells. We speculate, however, that a five-hour incubation with test compounds cannot account for this substantial increase. Most importantly, we did not detect centrosome fragmentation in normal primary HMEC treated with CCCI-01. This suggests that this compound targets a mechanism that BT-549 cells, but not healthy primary cells, rely on for the maintenance of centrosome integrity.

### Potential mechanisms of action of CCCI-01

CCCI-01 does not seem to induce obvious centrosome abnormalities in interphase BT-549 cells (Supplemental Figure 4). In contrast, Griseofulvin is known to bind to tubulin [14-16] and to inhibit centrosome coalescence in interphase cells [12]. Interphase cells treated with 10 μM CCCI-01, which resulted in over 90 % of de-clustering in mitotic cells, typically contained only one centrosome dot per cell, similar to DMSO control. Increasing the concentration to 20 μM or 40 μM did not seem to alter centrosome organization of interphase cells (Supplemental Figure 4). This suggests that CCCI-01 inhibits centrosome clustering in a mitosis-specific manner. CCCI-01 may target a protein that is highly expressed during mitosis or has a mitosis-specific function. Concentrations higher than 30 μM caused severe cell detachment from the substratum within 5 hours, indicating general cytotoxicity of this compound at these higher concentrations.

Our MTT assay, clonogenic assay and CFC assay showed that the nitrofuramide CCCI-01 selectively affected the survival and/or proliferation of several types of cancer cells while sparing normal cells. The apoptosis assay results further demonstrated that apoptosis was increased in BT-549 but not in MCF-10A cells by CCCI-01. These data indicate that this class of small molecules, especially CCCI-01, is selectively cytotoxic in cancer cells. Based on our flow cytometry analysis (Figure 4C), cytotoxicity of CCCI-01 is likely to be brought on by a combination of cell death and cell cycle arrest. Our structure-activity relationship analysis suggests that cytotoxicity caused by CCCI-01, 02 and 03 is due to centrosome de-clustering and that the spacing between the 5-nitro-2-furamide, a common structure in these compounds, and a 5- or 6-membered ring may be important for their activity (Figure 7).

### Centrosome de-clustering and mitotic arrest

All of our active compounds increased mitotic indices, confirming previous reports that centrosome de-clustering often results in mitotic arrest [6-8, 12]. It has been reported that spindle assembly checkpoint halts entry to anaphase until adequate spindles are established in cells with supernumerary centrosomes [6]. Depletions of certain proteins, however, do not arrest cells in mitosis while inducing multipolar spindle formation [7]. Some of these proteins are chromosomal passenger proteins, Aurora-B, Borealin, INCENP and Survivin [7]. Since our active compounds prolonged mitosis, it is unlikely that their targets are these chromosomal passenger proteins.

Through our high-content screen, we identified 14 novel compounds that inhibit centrosome clustering or induce centrosome fragmentation. One of the active compounds was examined in detail and showed selective cytotoxicity to cancer cells over primary normal cells. This study is an initial step in the development of various classes of drugs that block centrosome clustering through different pathways with opportunities for improving their activity and specificity through medicinal chemistry. Future studies will be aimed at identifying potential cellular targets of CCCI-01, 02 and 03 and determining the *in vivo* pharmacokinetic properties, toxicity, and efficacies of these compounds in models on tumor growth. The studies represented here provide a promising novel approach to the development of cancer-specific therapy and understanding fundamental molecular mechanisms of bipolar mitosis.

## METHODS

### Cell lines

BT-549 cells were cultured in RPMI with 10% fetal bovine serum (FBS, Life Technologies, Carlsbad, CA, USA) and 0.023 IU/ml insulin (Sigma-Aldrich, St Louis, MO, USA). MDA-MB-231 and A-549 cells were cultured in DMEM (Life Technologies) supplemented with 10% FBS. HCT-116 cells were cultured in McCoy's 5a (Life Technologies) with 10% FBS. MCF-10A cells were cultured according to Imbalzano *et al*. [32]. All cell lines were obtained from ATCC.

### Primary human mammary epithelial (HMEC) cells

Ten histologically normal discard tissue samples from healthy women undergoing reduction mammoplasty surgery were collected with informed consent approved by the University of British Columbia Research Ethics Board. Single mammary cells were prepared [33, 34] and proliferative epithelial cells were enriched using a 3-day pre-culture method described previously (Supplemental Figure 5) [35]. Cells were trypsinized and viable HMECs isolated by FACS were cultured in DMEM/F12 supplemented with 10 ng/ml epidermal growth factor, 10 ng/ml cholera toxin, 1 μg/ml insulin, 0.5 μg/ml hydrocortisone and 5% FBS, overnight, prior to compound treatment.

### Small molecules

All compounds used in this study were purchased from Maybridge (Cambridge, UK). Stock solutions in DMSO were stored at -20 °C.

### Sample preparation for high-content screening

BT-549 cells were cultured in 96-well plates suitable for fluorescence analysis (Perkin Elmer, Waltham, MA, USA), overnight. Test compounds were delivered to each well using a robotic pinning instrument that gave a final concentration of approximately 17 μM of each compound. Cells were incubated with small molecules for five to seven hours, fixed with pre-warmed 4 % paraformaldehyde with 0.1 % triton X-100 in PBS for 15 minutes, and then incubated with 1 % BSA (Sigma-Aldrich) in PBS for 30 minutes. Mouse TG-3 [20] and rabbit anti-pericentrin (AbCam, Cambridge, UK) were applied to the samples (1/200 and 1/2000 in PBS, respectively) overnight at 4 °C. Secondary antibodies (goat anti-mouse Alexa 568 and goat anti-rabbit Alexa 488, Life Technologies) were incubated with cells (1/500 in PBS) for one hour at room temperature. Cells were stained with 500 ng/ml Hoechst 33342 (Life Technologies) for 10 minutes. Immunolabelled samples were stored in PBS at 4 °C until further examination.

For secondary screening, a dilution series of compounds as well as corresponding concentrations of DMSO was examined in duplicates. Cells were processed as described above.

### Image acquisition and data analysis

A Cellomics Array Scan VTI (Thermo Scientific, Waltham, MA, USA) fluorescent imaging system equipped with a Caliper robotic plate loader (PerkinElmer) was used for automated image acquisition. Fifteen fields with three channels, Hoechst for DNA stain, TG-3 for mitotic cell detection, and pericentrin for centrosome detection, were imaged for each well using a 10x objective. Each field was auto-focused to ensure clear image acquisition.

For data analysis, Thermo Scientific Compartmental Analysis algorithm (Thermo Scientific) was employed. Cell nuclei were identified (Figure 1A) and a border was introduced by expanding 5 pixels outward from the nuclear boundary to define a region of interest (ROI). If the mean pixel intensity of TG-3 labeling within the ROI was above a threshold, the cell was considered to be in mitosis (blue circles for nuclear boundary of mitotic cells and green circles for ROI in Figure 1B and C). Those cells below the threshold were considered non-mitotic and the nuclear boundary was drawn in orange (Figure 1A-C). The number of pericentrin foci within the ROI (light blue dots, Figure 1C) was automatically counted. When centrosome foci within the ROI were more than two, the cell was considered to have de-clustered centrosomes. The percentage of mitotic cells with de-clustered centrosomes was determined for each treatment. In addition, mitotic index (% of cells in mitosis) was obtained by dividing the number of TG-3 positive cells by the total number of nuclei identified in the Hoechst channel.

### Immunofluorescence microscopy under standard culture conditions

Cells were grown on coverslips in 6-well plates overnight, treated with compounds for 5 hours, fixed with cold methanol for 10 minutes, and subjected to immunolabeling according to Dobreva *et al*. [36]. Primary antibodies used were mouse DM1A anti-α-tubulin (Sigma-Aldrich) and rabbit anti-pericentrin (Abcam, Cambridge, MA, USA) at 1/1000 and 1/2000, respectively. Goat anti-mouse Alexa 594 and goat anti-rabbit Alexa 488 (Life Technologies) were applied at 1/400 as secondary antibodies. Cells were mounted in ProLong Gold mounting media (Life Technologies).

Specimens were imaged and analyzed under an epifluorescence microscope, Colibri (Carl Zeiss, Oberkochen, Germany). Images were acquired with an AxioCam HRc camera and AxioVision 3.1 software (Carl Zeiss), and brightness/contrast was adjusted using ImageJ (NIH).

Centrosome de-clustering was analyzed based on Fielding *et al*. [8]. Centrosome number and arrangement, spindle polarity, and chromosome alignment were examined, and the frequency of mitotic cells with de-clustered centrosomes was determined.

### The MTT assay

BT-549, MDA-MB-231, A-549, HCT-116, MCF-10A cell lines and freshly isolated normal primary HMECs (passage 0) were examined. Cells (2,000 cells/90 μl/well) were plated in 96-well plates for several hours to allow adhesion to the plates for established cell lines. Primary HMEC were cultured overnight in 96-well plates (5,000 cells/90 μl/well) following isolation by FACS. A dilution series of compounds was prepared with appropriate media, added to the cell culture (10 μl/well) and incubated for two days.

For the MTT assay, 10 μl of 5 mg/ml thiazolyl blue tetrazolium bromide in PBS was applied to each well and incubated for four hours. 100 μl of solubilization solution (10% SDS and 0.01N HCl) was added and the plates were kept in the incubator overnight. OD were measured at 570 nm and 660 nm by a spectrophotometer. A_570_ was subtracted from A_660_, and the values were normalized to the negative control without DMSO.

### Cell cycle and cell death analyses using flow cytometry

BT-549 cells were cultured with 5 μM CCCI-01 or 0.05% DMSO, and harvested by trypsinization at varying time points. After a PBS wash, suspended cells were fixed and stored in 70% ethanol at 4 °C. Cells were stained with 20 μg/ml Propidium Iodide with 0.1 % TritonX-100 and 200 μg/ml DNase-free RNase A (Sigma-Aldrich) in PBS. FACS Calibur (BD Biosciences, San Jose, CA, USA) with a 488nm laser and a 585/42 filter was used for the detection of Propidium Iodide that stained DNA. 3,000 events were collected for each sample. Doublets were eliminated based on their distribution in area vs. width plots for Propidium Iodide signal, and the remaining events as area were analyzed for cell cycle and apoptosis using FlowJo (Tree Star Inc., Ashland, OR, USA).

### Apoptosis assay

Quantification of cytoplasmic nucleosomes was carried out using Cell Death Detection ELISA (Roche, Basel, Switzaland). BT-549 and MCF-10A cells were seeded at 1.5 × 10^5^ cells/well in 6-well plates. Cells were let to adhere for several hours, and then treated with a dilution series of CCCI-01. The highest concentration of DMSO (0.1 %) applied for CCCI-01 treatment was included to assess the solo effect of DMSO. After 19 hours of incubation, cells were spun down at 200 g for 10 min, and processed for cytoplasmic nucleosome detection according to the manufacturer's instruction.

### Clonogenic assay

Clonogenic assays were carried out according to Franken *et al*. [22]. In brief, BT-549 and MCF-10A cells were trypsinized to obtain single cell suspensions and seeded at 100 and 50 cells/well in 6-well plates, respectively. Cells were let to adhere for several hours, and CCCI-01 or DMSO was applied. All treatments with CCCI-01 received 0.06 % of DMSO. After 9 days of incubation, cells were fixed and colonies consisting of more than 50 cells were enumerated for each treatment.

### The CFC assay using primary normal bone marrow cells

Bone marrow samples were collected from the iliac crest of healthy donors with informed consent and the procedure was approved by the University of British Columbia Research Ethics Board. Mononuclear cells were isolated with Ficoll-Hypaque (Sigma-Aldrich) density gradient separation and CD34+ cells were selected by EasySep CD34 positive selection kit (STEMCELL Technologies, Vancouver, Canada). 10,000 monoculear cells and 2,000 cells selected for CD34+ were plated in methylcellulose media (MethoCult H4330 with EPO, STEMCELL Technologies) supplemented with cytokines [37] and varying concentrations of CCCI-01 in duplicates, and were cultured for 16 and 15 days, respectively. All samples contained 0.1% DMSO, except for 0 μM treatment that did not receive any DMSO. Colonies were evaluated according to the manufacture's instruction for MethoCult (STEMCELL Technologies).

## Supplementary Figures and Tables




