# Supplementary material for: Identification of novel small molecule inhibitors of centrosome clustering in cancer cells
Source: Oncotarget. 2013 Sep 25;4(10):1763–76. doi: 10.18632/oncotarget.1198 (PMC3858562; doi:10.18632/oncotarget.1198)
Supplement: Supplementary file 1 [file oncotarget-04-1763-s001.pdf]

Identification of novel small molecule inhibitors of centrosome clustering in cancer cells - Kawamura et al

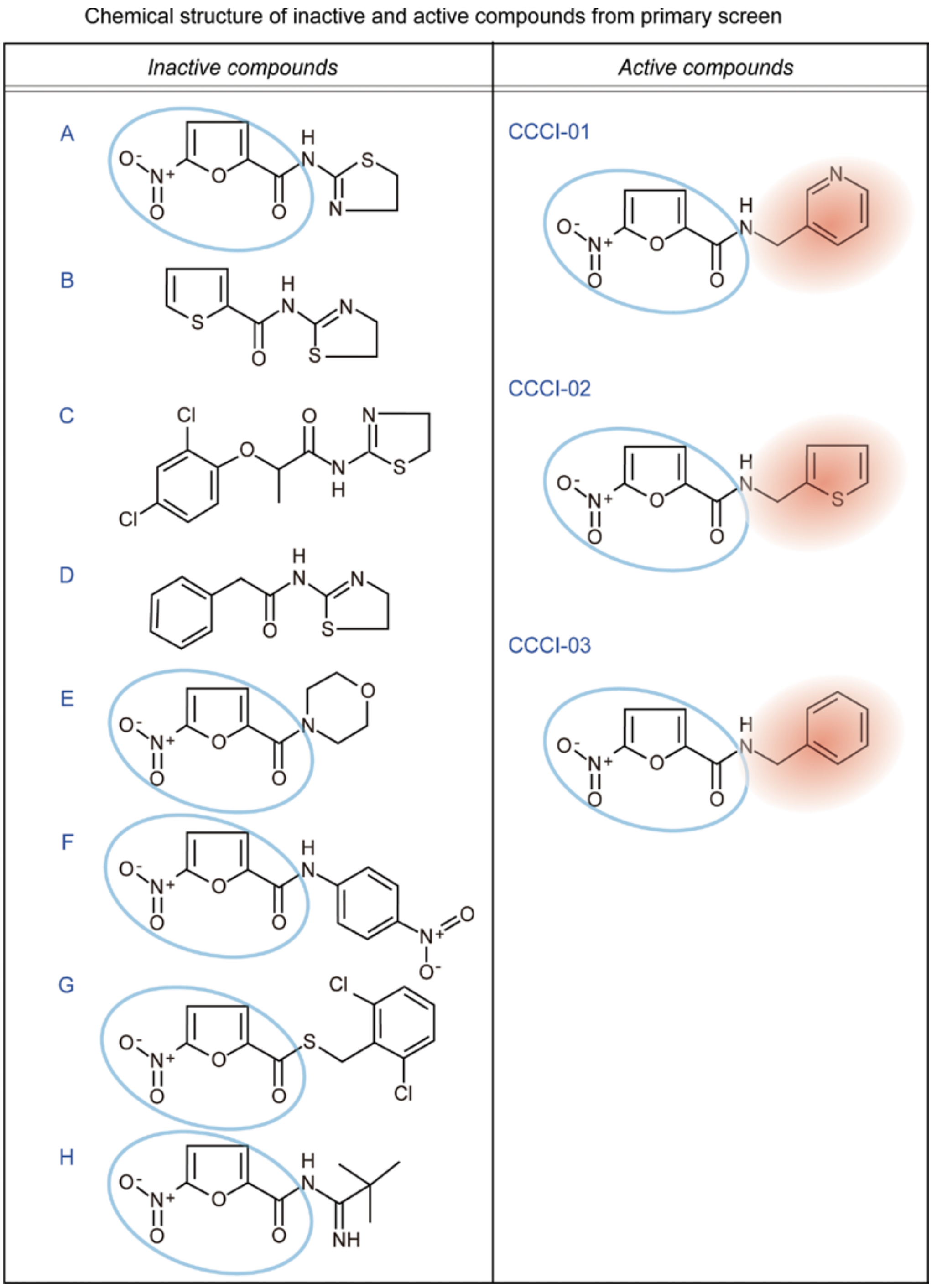

Supplemental Figure 1: Chemical structures of compounds that were similar to CCCI-01, 02 and 03, but resulted negative in the primary screen.

Compounds that did not show centrosome de-clustering activities in the primary screen and were similar to CCCI-01, 02 and 03 (right column) are shown in the left column. A chemical structure in common between active and inactive compounds is circled in blue. Red highlights indicate an extra carbon and ring that is only present in the active compounds.

A is N2-(4,5-dihydro-1,3-thiazol-2-yl)-5-nitro-2-furamide.

B is N2-(4,5-dihydro-1,3-thiazol-2-yl)thiophene-2-carboxamide.

C is N1-(4,5-dihydro-1,3-thiazol-2-yl)-2-(2,4-dichlorophenoxy)propanamide.

D is N1-(4,5-dihydro-1,3-thiazol-2-yl)-2-phenylacetamide.

E is morpholino(5-nitro-2-furyl)methanone.

F is N2-(4-nitrophenyl)-5-nitro-2-furamide.

G is 2,6-dichlorobenzyl 5-nitrofuran-2-carbothioate.

H is N2-(2,2-dimethylpropanimidoyl)-5-nitro-2-furamide.

The MTT assay of CCCI-01, 02, 03 and DMSO

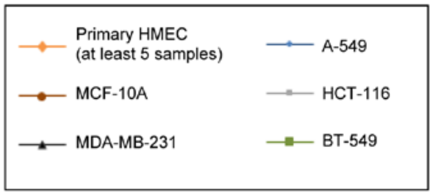

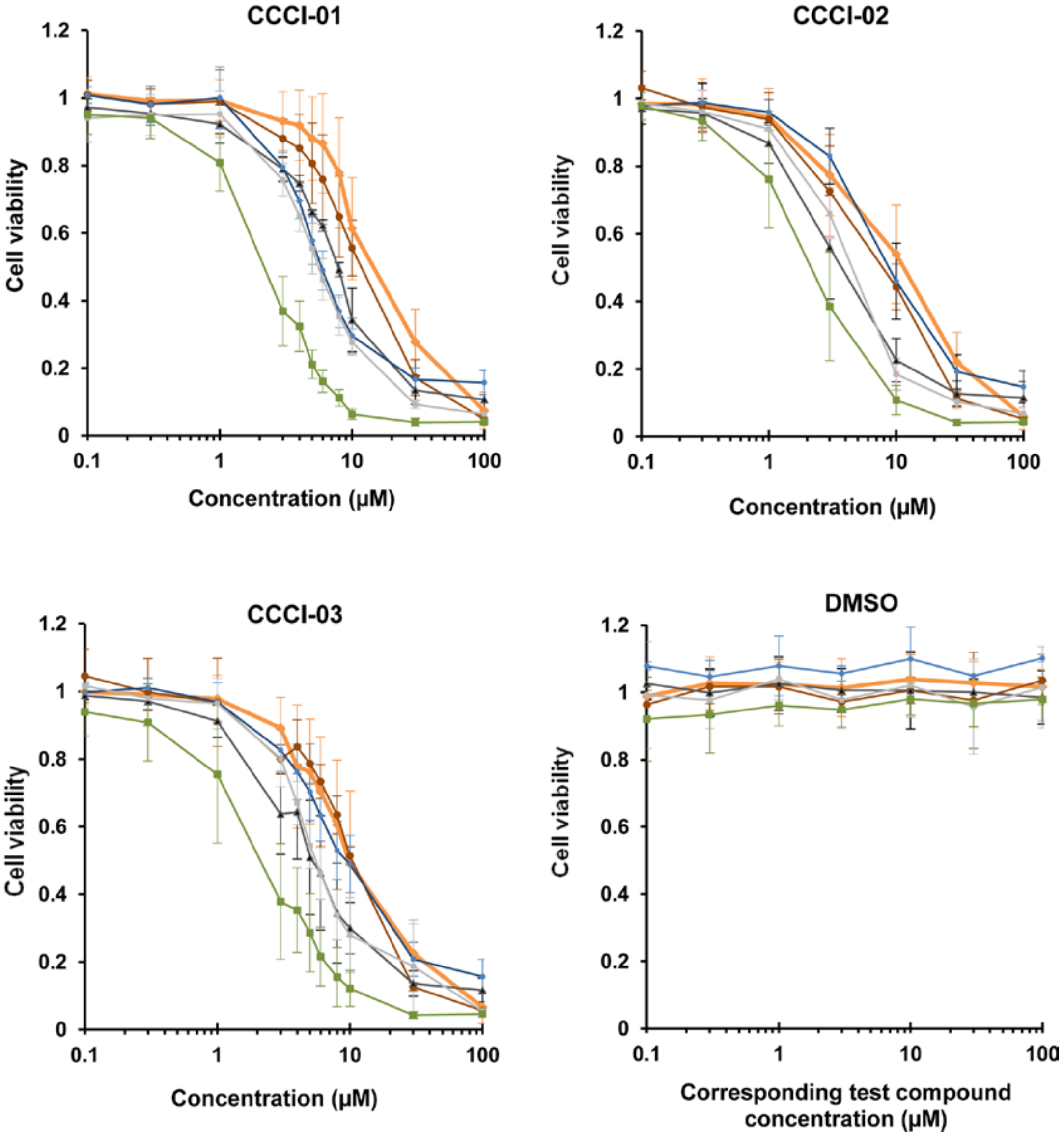

Supplemental Figure 2: The MTT assay of CCCI-01, 02, 03 and DMSO

The MTT assay of CCCI-01, 02 and 03 to examine their cytotoxicity in normal primary HMEC, MCF-10A, BT-549, MDA-MB-231, A549, and HCT-116. DMSO had little effect on cell viability at all the concentrations examined. CCCI-01 showed the most promising differential effects between cancer and normal cells.

CCCI-01) BT-549, $P < 0.001$ at 1-30 µM; MDA-MB-231, $P < 0.05$ at 1 µM, 4-8 µM, $P < 0.005$ at 3, 10 and 30 µM; A-549, $P < 0.02$ at 3-10 µM; HCT-116, $P < 0.02$ at 3-10 µM, student's t-test compared with normal primary HMEC.

CCCI-02) BT-549, $P < 0.02$ at 1 and 30 µM, $P < 0.0002$ at 3 and10 µM; MDA-MB-231, $P < 0.05$ at 3, 30 and 100 µM, $P = 0.0002$ at 10 µM; HCT-116, $P < 0.02$ at 10 and 30 µM, student's t-test compared with normal primary HMEC.

CCCI-03) BT-549, $P < 0.03$ at 1 and 4 µM, $P < 0.005$ at 3 and 5-30 µM; MDA-MB-231, $P = 0.001$ at 10 µM, $P = 0.028$ at 30 µM, student's t-test compared with normal primary HMEC.

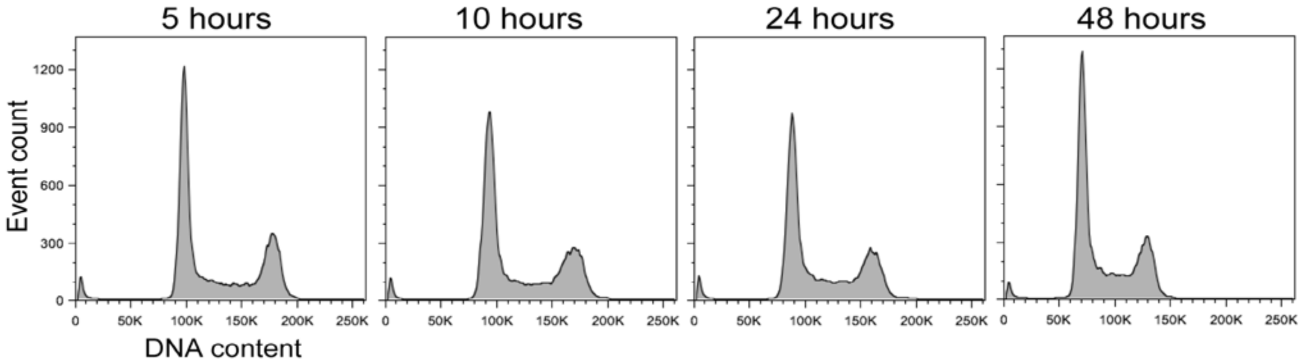

Supplemental Figure 3: Cell cycle and cell death analyses by flow cytometry in the DMSO negative control

The distributions of all populations are essentially the same in all time points examined for DMSO control.

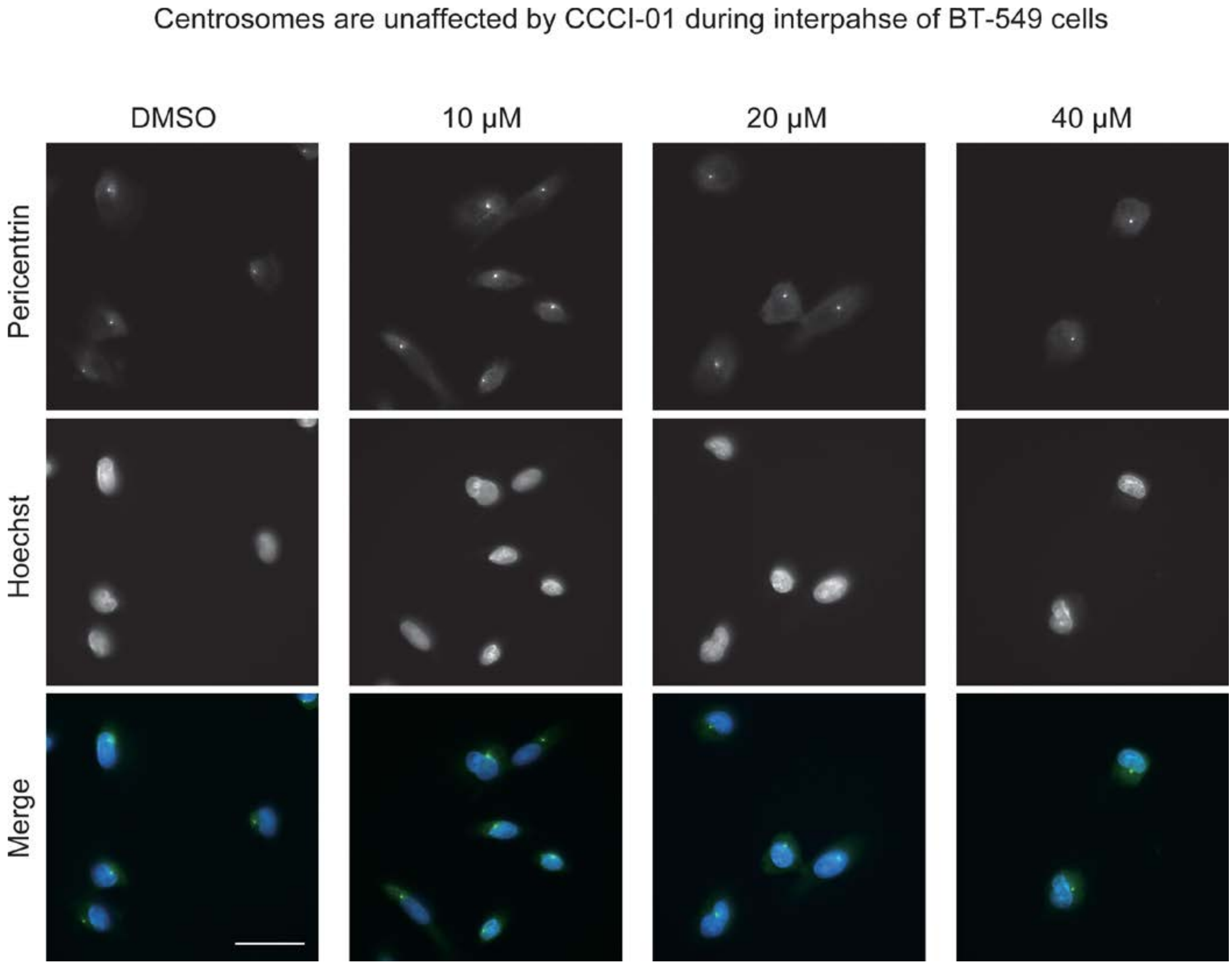

Supplemental Figure 4: Centrosome arrangement in interphase of BT-549 treated with CCCI-01

Immunofluorescent labeling of pericentrin and DNA stain in BT-549 cells treated with CCCI-01 for 5 hours. At 10 µM, which resulted in over 90 % of de-clustered mitotic cells, the centrosome organization in interphase was similar to DMOS control, displaying typically one centrosome dot per cell. Increasing the concentration of CCCI-01 to 40 µM did not seem to alter centrosome arrangement in interphase cells. This indicates that CCCI-01 disrupts centrosome integrity specifically during mitosis. In the merged images, pericentrin is shown in green, Hoechst staining is in blue. Bar = 50 µm.

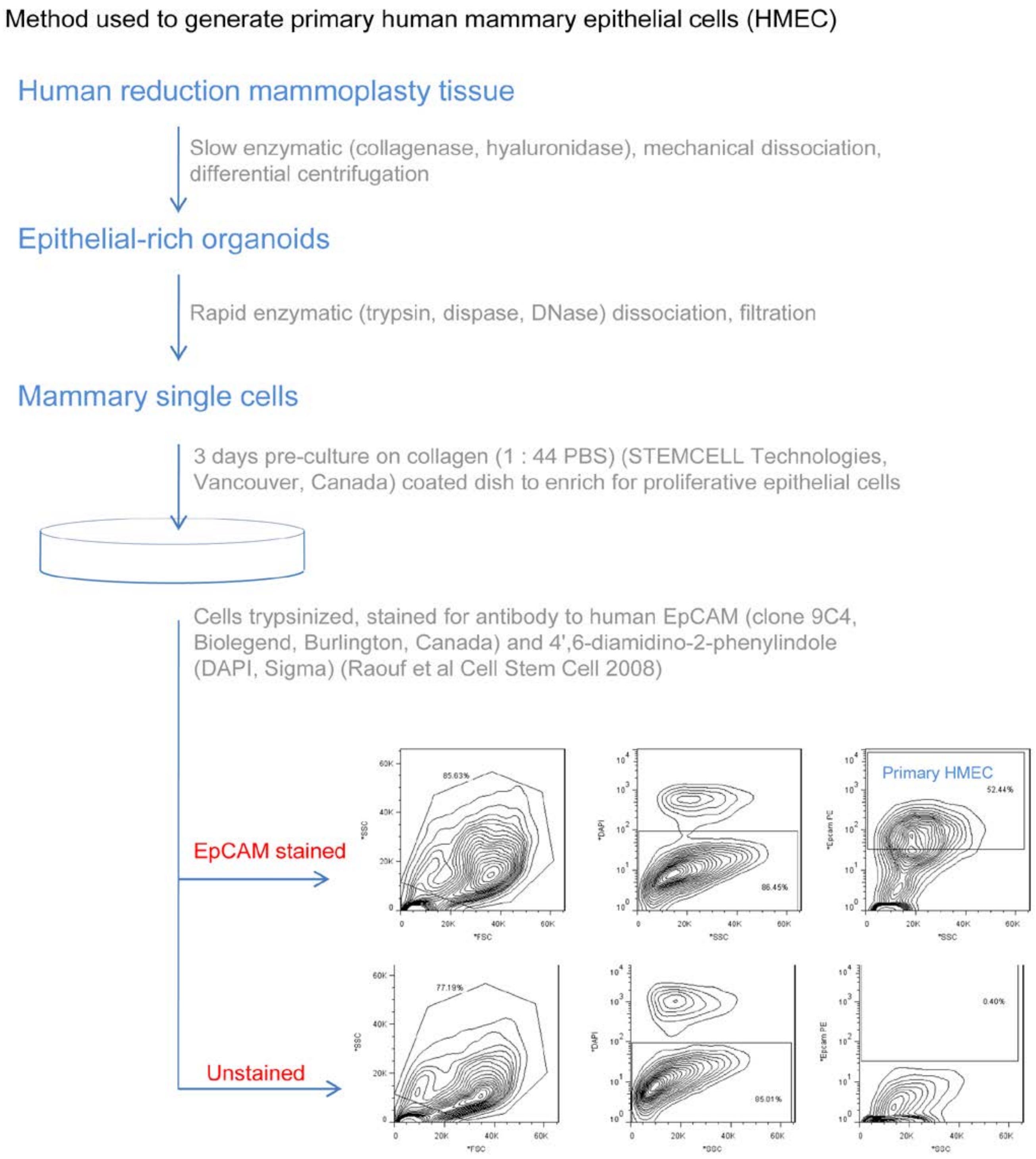

Supplemental Figure 5: Schematic illustration and details of method for primary HMEC isolation

Detailed information on the method used to isolate normal primary HMEC.
