# Supplementary material for: Identification of novel small molecule inhibitors of centrosome clustering in cancer cells
Source: Oncotarget. 2013 Sep 25;4(10):1763–76. doi: 10.18632/oncotarget.1198 (PMC3858562; doi:10.18632/oncotarget.1198)
Supplement: Supplementary file 2 [file oncotarget-04-1763-s002.pdf]

Identification of novel small molecule inhibitors of centrosome clustering in cancer cells - Kawamura et al

Supplemental Table 1: Results of CFC assays for each sample

Values are average ± s.d. of duplicates for each treatment. Values are normalized to the DMSO control.

	Unselected normal bone marrow cells		Normal bone marrow cells selected for CD34+	
	CFC-G/M	BFU-E/CFU-E	CFC-G/M	BFU-E/CFU-E
DMSO	1.00 ± 0.33	1.00 ± 0.24	1.00 ± 0.07	1.00 ± 0.01
0 µM	1.13 ± 0.15	1.32 ± 0.02	1.08 ± 0.10	1.13 ± 0.05
0.3 µM	0.92 ± 0.15	1.08 ± 0.07	0.92 ± 0.03	1.04 ± 0.11
1 µM	1.13 ± 0.29	1.00 ± 0.24	1.15 ± 0.02	1.44 ± 0.01
3 µM	0.49 ± 0.18	1.12 ± 0.07	1.22 ± 0.07	1.27 ± 0.12
10 µM	0.31 ± 0.00	0.2 ± 0.09	0.99 ± 0.09	0.85 ± 0.23
